# Acidic Microenvironment–Sensitive Core-Shell Microcubes: The Self-assembled and the Therapeutic Effects for Caries Prevention

**DOI:** 10.1055/s-0042-1757464

**Published:** 2022-12-19

**Authors:** Tsai-Miao Shih, Jui-Fu Hsiao, Dar-Bin Shieh, Guochuan Emil Tsai

**Affiliations:** 1 Department of Research and Development, SyneuRx International (Taiwan) Corp., New Taipei City, Taiwan; 2 School of Dentistry and Institute of Oral Medicine, National Cheng Kung University, Tainan, Taiwan; 3 Center of Applied Nanomedicine and Core Facility Center, National Cheng Kung University, Tainan, Taiwan; 4 Department of Stomatology, National Cheng Kung University Hospital, Tainan, Taiwan; 5 Department of Psychiatry and Biobehavioral Sciences, UCLA School of Medicine, Los Angeles, California, United States

**Keywords:** probiotic-preferable environment, caries, environment-sensing, sodium fluoride, rebalance

## Abstract

**Objectives** The aim of this study was to develop a new material with integrated interface design that could achieve the purpose of environmental-sensing controlled release against cariogenic bacteria. Furthermore, this material can rebalance oral flora and serve as a preventive and reparative measure of dental caries.

**Materials and Methods** NaF@PAA@HA@polyelectrolytes@HA@PAA particles were synthesized using the method of two-solution phases precipitation followed by biocompatible polymers coating layer by layer. The structure of the particles was confirmed by transmission electron microscope. The fluoride release profile was measured by fluoride ion electrode. Antimicrobial activity against the cariogenic microorganisms was analyzed by scanning electron microscopy and energy dispersive spectrum. The efficacy experiments were conducted on tooth enamel slides to evaluated fluoride absorption and antibacterial activity of the prototype toothpaste containing microcube particles

**Results** The structure of NaF@PAA@HA@polyelectrolytes@HA@PAA particles showed a core surrounded by tooth-adhesion polymer layers in thin fin or filament structure. The loaded concentration of fluoride in the particles' core was 148,996 ± 28,484 ppm. NaF@PAA@HA@polyelectrolytes@HA@PAA particles showed selective inhibition of cariogenic microorganisms over probiotic strains and stronger fluoride adhesion on tooth enamel. A burst release (over 80%) of fluoride from the particle-containing toothpaste was observed under cariogenic acidic environment (pH < 5), while it remained extremely low under neutral environment. Compared with the best results of commercial toothpastes, our prototype toothpaste increased enamel fluoride uptake by 8-fold in normal enamel slides and by 11-fold in the slides with induced white spot lesions after either 1- or 7-day treatment. The prototype toothpaste also showed better inhibition of cariogenic microorganisms than the commercial brands. The coverage area of cariogenic bacteria under our toothpaste treatment was 73% on normal enamel slides compared with the commercial brands, while it was 69% in the induced white spot lesions.

**Conclusions** In our study, an intelligent toothpaste was developed that selectively inhibits cariogenic bacteria by microenvironment proton-triggered fluoride release. Such novel design would accomplish a favorable flora balance for optimal long-term oral health.

## Introduction

Dental caries is one of the most prevalent human infectious diseases. Effective caries prevention, however, remains an unmet clinical need around the globe. 1 The combination of four major factors leads to caries formation: dietary carbohydrates, cariogenic flora, tooth, and time. 2 Cariogenic bacteria produce glucosyltransferases (GTFs) and synthesize water-insoluble extracellular polysaccharides from sucrose that assists bacteria adhesion to tooth surface and facilitates the formation of dental biofilms. 3 Acidogenic and aciduric bacteria such as *Streptococcus mutans* further metabolize carbohydrate to organic acids, thus creating a low-pH microenvironment in the biofilm that causes dissolution of the tooth mineral components and dental caries eventually. 1 4

The most common measure to decrease the prevalence and severity of dental caries is the use of fluoride. 5 Many studies have shown that fluoride inhibits acid production and GTFs' activity of *S. mutans* while enhance the acid tolerance of teeth. 6 Investigators have also shown that fluorapatite nanocrystals also harbor such advantages. 7 It is well known that bacteria living in biofilms usually show increased resistance to various antimicrobial agents and better survive the environmental insults. 8 Hence, fluoride must be present in the correct place (biofilm fluid or saliva) and time (during sugar exposure) to protect against caries. 9

The method of layer-by-layer formation is an emerging and important methodology in the fabrication of polymeric nanoparticles because of controllable surface chemistry and ease of industrial mass production. 10 11 Hydroxyapatite (HA; Ca _10_ (PO _4_ ) _6_ (OH) _2_ ) nanoparticles have attracted much attention as an excellent biomaterial, especially in tissue engineering and drug delivery systems. 12 13 These nanoparticles can provide additional advantages for effective caries prevention as a result of their extreme small size and environmentally controlled release. Small HA nanoparticles could promote dental plaque penetration as well as selectively inhibit the cariogenic bacteria in the microenvironment. In this study, we developed a particle with sodium fluoride nanocrystal core and a shell of polyelectrolytes–HA hybrid layer with tooth adhesive polymeric nano-brushes. The brush shell–fluoride core particle forms self-assembling protective layers upon exposure to tooth surface and selectively inhibits cariogenic pathogens while preserving probiotics that provide a novel caries prevention strategy.

## Materials and Methods

### Synthesis of NaF@PAA@HA@polyelectrolytes@HA@PAA Particles

To synthesize HA nanoparticles, H _3_ PO _4_ (100 mL, 0.6 M) solution was added dropwise into Ca(OH) _2_ solution (125 mL, 0.8 M) and stirred at 800 rpm and 80°C for 2 hours. Simultaneously, the pH value was adjusted to 10 to 11 with ammonia hydroxide solution. Then, the reaction solution was aged for 24 hours at 70°C and precipitated. Afterward, the white precipitate was rinsed by deionized water until the pH returned to 7. After centrifugation at 12,000 rpm for 5 minutes at 25°C (Kubota Co., Ltd), the precipitate was dried in vacuum oven at 70°C for 24 hours to obtain HA nanoparticles. 14

**Fig. 1:**
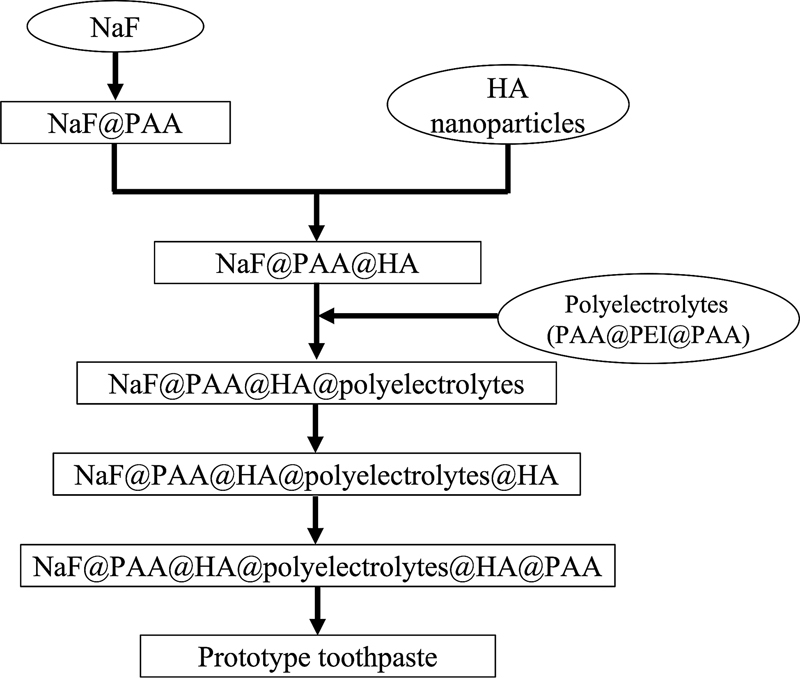
The flowchart of synthesis process of NaF@PAA@HA@polyelectrolytes@HA@PAA particles.

To synthesize NaF@PAA@HA@polyelectrolytes@HA@PAA particles ( Fig. 1 ), saturated aqueous solution of NaF (5 mL) was slowly added into anhydrous alcohol (50 mL) under stirring at 400 rpm and 25°C for 1 hour to form the functional cores. Then, 5 mL of 1 mM polyacrylic acid (PAA; molecular weight [MW], 5,000 kD) was mixed with the functional cores under stirring at 600 rpm for 2 hours to form NaF@PAA particles, and the particles were collected by centrifugation at 12,000 rpm for 5 minutes. Next, NaF@PAA particles and HA nanoparticles were mixed and stirred at 400 rpm for 1 hour to synthesize NaF@PAA@HA particles, which were collected by centrifugation at 12,000 rpm for 5 minutes. Subsequently, 5 mL of 1 mM PAA and polyethyleneimine (PEI; MW, 1,800 kD) were added sequentially into NaF@PAA@HA particles under stirring at 600 rpm for 2 hours, respectively. The above steps were repeated to form NaF@PAA@HA@polyelectrolytes@HA (the polyelectrolytes were PAA@PEI @PAA multiple layers 15 ) and NaF@PAA@HA@polyelectrolytes@HA@PAA particles.

### Characterization of NaF@PAA@HA@Polyelectrolytes@HA and NaF@PAA@HA@Polyelectrolytes@HA@PAA Particles

The morphology and particle size were observed and determined by transmission electron microscope (TEM; JEOL, JEM-2100F) at ×10,000 magnification. The samples were prepared on the copper grids at the concentration of 10 μg/mL.

### Fluoride Concentration of NaF@PAA@HA@Polyelectrolytes@HA@PAA Particles

The fluoride concentration of NaF@PAA@HA@polyelectrolytes@HA@PAA particles was evaluated by fluoride ion electrode (Cole-Parmer Co., Cat. No. 27502–19). The particles were suspended in 0.1-N HCl at 1 mg/mL to detect total fluoride concentration. 16 The solution was mixed with equal volume of total ionic strength adjuster buffer. The fluoride ion electrode was placed into the mixture to measure the fluoride release. A standard curve for NaF concentration was made by serial dilutions of NaF solution with known concentration.

### Bacterial Culture

*S. mutans* (American Type Culture Collection [ATCC] 25175) and *Streptococcus gordonii* (ATCC 35105) were obtained from the Food Industry Research and Development Institute in Hsinchu, Taiwan. *S. mutans* were cultured in tryptic soy broth (Becton Dickinson Bacto) and *S. gordonii* were cultured in brain–heart infusion broth (Becton Dickinson Bacto). Both strains of bacteria were cultured at 37°C in aerobic conditions. Due to the necessity of metabolism, bacteria were switched to fresh broth supplemented with 1% sucrose and glucose when performing antibacterial experiment. 17

### Antibacterial Activity of NaF@PAA@HA@Polyelectrolytes@HA@PAA Particles

Prior to the experiment, the NaF@PAA@HA@polyelectrolytes@HA@PAA particles were sterilized by UVC light for 15 minutes and resuspended in sterilized deionized water. Both *S. mutans* and *S. gordonii* were grown to log phase and diluted to the optical density at 600 nm (OD _600_ ) of 0.01 with respective broth. NaF@PAA@HA@polyelectrolytes@HA@PAA particles at 1 mg/mL were added to respective bacterial cultures, and the cultures were incubated at 37°C for 8 hours. The cultures with no added synthesized particles were served as controls. After incubation, all samples were diluted to 10 ^−4^ to 10 ^−6^ folds with broth containing 1% sucrose and glucose. The antibacterial activity was evaluated by colony-forming unit assay, as described. 18

### Generation of Artificial White Spot Lesions on Human Teeth

The normal human teeth, excluding those with any detectable flaws, were selected and the attached soft tissue debris was removed. The cleaned teeth were then coated with acid-resistant nail varnish and left a 5 × 3 mm ^2^ window for the generation of white spot lesions. These teeth were immersed in the demineralization solution at 37°C for 4 days, with the solution changed daily to produce lesions. 19 The demineralizing solution contained 2.2-mM CaCl _2_ , 2.2-mM NaH _2_ PO _4_ , and 50-mM acetic acid, with pH 4.4 adjusted by 1-M KOH. The study protocol was approved by the Institutional Review Board of National Cheng Kung University Hospital (approval number ER-101–035).

### Fluoride Absorption of NaF@PAA@HA@Polyelectrolytes@HA@PAA Particles on Tooth Enamel

The tooth enamel slides with induced white spot lesions and the clean slides were brushed with 10 mg NaF@PAA@HA@polyelectrolytes@HA@PAA particles for 30 seconds and then shaken at 30 rpm in artificial saliva for 4 hours. This procedure was repeated thrice a day. After the treatments, both experimental and controlled slides were divided into two groups: one group was soaked in 1-mL 0.1-N HCl to detect the fluoride concentration, while another group was fixed and dehydrated for further analyses by scanning electron microscopy (SEM) and energy dispersive spectrum (EDS; JSM-7000 JEOL Co., Ltd). For SEM, the tooth enamel slides were put on the double-coated carbon conductive tape and coated with platinum using autofine coater for better electrical conduction. The ratio of fluoride absorption onto tooth enamel slides was quantified by EDS.

### Preparation of Prototype Toothpaste and Commercial Toothpastes

The prototype toothpaste was composed of 50-g HA, 10-mL glycerin, 20-mL lauryl alcohol polyethylene glycol ether and laureth 4, 1.75-g methyl cellulose, 2-mL sodium lauroyl sarcosinate, 0.02-g methyl paraben, 1-mL mineral oil, 1-g NaF@PAA@HA@polyelectrolytes@HA@PAA particles, and 14.23-mL water. To compare the inhibition efficacy with the prototype toothpaste, we chose four commercial fluoride-containing toothpastes: Colgate Total Pro Gum Health toothpaste (brand A), Sensodyne Fluoride toothpaste (brand B), Whitemen Double Fluoride and Calcium toothpaste (brand C), and Biorepair Total Protective Repair toothpaste (brand D).

### Fluoride Release of the Prototype Toothpaste

The prototype toothpaste at 100 mg/mL was suspended in artificial saliva for 7 days at pH 5 and pH 7, respectively. On day 1 and day 7, samples at each time points were collected for 1 mL (every hour before 12 hours, and then every 6 hours till 24 hours). Subsequently, fluoride release of the collected samples was evaluated by fluoride ion electrode. The prototype toothpaste was suspended in 0.1-N HCL at 100 mg/mL to determine the total fluoride concentration. The releasing profile was presented as the percentage of release over time by the following equation: ratio (%) = F concentration _sample_ / F concentration _total_ × 100%.

### Fluoride Absorption on Tooth Enamel after Treatment with Toothpastes

The clean tooth enamel slides and the slides with induced white spot lesions were brushed with 100 mg of prototype or commercial toothpaste for 30 seconds and shaken in artificial saliva for 4 hours. This procedure was repeated thrice a day. After either 1- or 7- day treatments, the ratio of fluoride absorption on controlled and experiment tooth enamel slides was quantified by SEM and EDS, respectively.

### Anticariogenic Activity of Toothpastes on Tooth Enamel

Both tooth enamel slides were brushed with 100 mg of prototype toothpaste, commercial toothpaste, or artificial saliva for 30 seconds. *S. mutans* was grown to log phase and diluted to a final concentration with OD _600_ of 0.01. The bacterial culture was then incubated with treated tooth enamel slides at 37°C for 4 hours. The above-mentioned steps were repeated thrice a day. After reaction for either 1 or 7 days, the tooth enamel slides were fixed and dehydrated for further analysis by SEM. The growth profiles of *S. mutans* on the treated tooth enamel slides were observed through SEM imaging. To quantify the antibacterial activity, the percentage of bacterial cover area of SEM images was evaluated by ImageJ software.

### Statistical Analysis

Student's *t* -test was applied for the studies on antibacterial activity, fluoride concentration, and fluoride absorption to determine the statistical significance.

## Results

### Characterization of NaF@PAA@HA@Polyelectrolytes@HA and NaF@PAA@HA@Polyelectrolytes@HA@PAA Particles

**Fig. 2:**
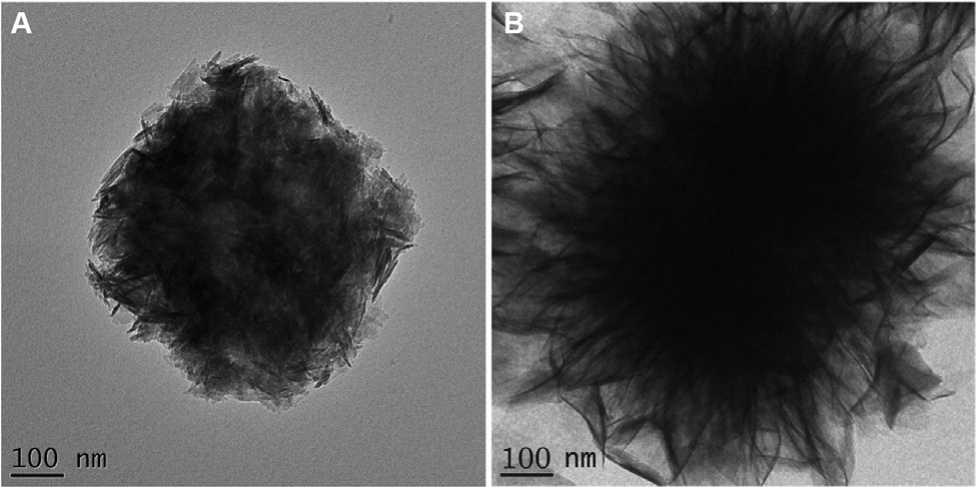
The TEM images of synthesized microparticles. ( **A** ) The structure of NaF@PAA@HA@polyelectrolytes@HA particles is showed as cubic shape of around 500 nm in size. ( **B** ) The filamentous structures extended from NaF@PAA@HA@polyelectrolytes@HA@PAA particles that served as adhesion layer to teeth surface.

The morphology of the core, NaF@PAA@HA@polyelectrolytes@HA, is illustrated in Fig. 2A . NaF@PAA@HA@polyelectrolytes@HA particles are cubic in shape, with size ranging from 300 to 1,000 nm. Fig. 2B shows the structure of NaF@PAA@HA@polyelectrolytes@HA@PAA. As illustrated, some filaments or thin fin structures are attached and extended outwardly from the outer surface of the core particles. The overall size of the NaF@PAA@HA@polyelectrolytes@HA@PAA particles ranges from 400 to 5,000 nm.

### Fluoride Concentration of NaF@PAA@HA@Polyelectrolytes@HA@PAA Particles

To verify that NaF@PAA@HA@polyelectrolytes@HA@PAA particles contained a sodium fluoride core, the particles were analyzed with the fluoride ion electrode. The mean value with standard deviation of fluoride concentration from five analyses was 148,996 ± 28,484 ppm, indicating the fluoride-containing particles were synthesized successfully.

### Differential Antibacterial Activity Assay of the Synthesized Particles (In Vitro)

**Fig. 3:**
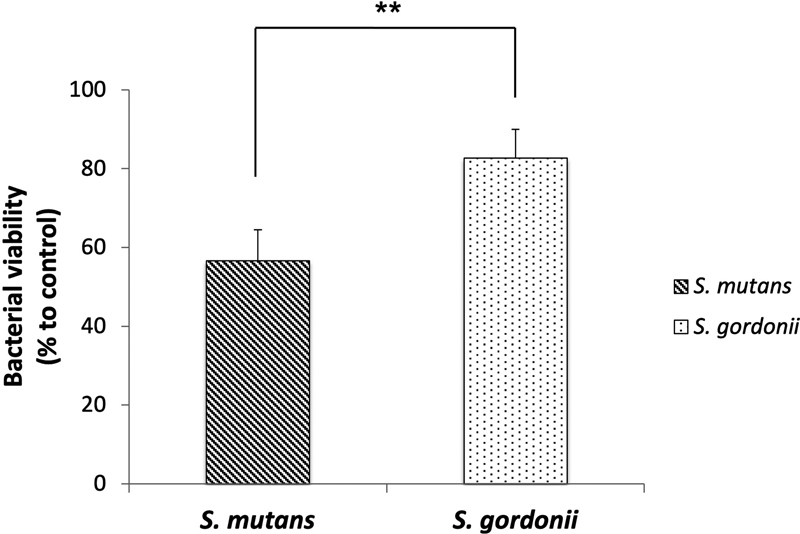
The antibacterial activities of NaF@PAA@HA@polyelectrolytes@HA@PAA particles to the pathogenic *S. mutans* and the probiotic *S. gordonii* . The particles showed preferential antibacterial activity toward pathogenic *S. mutans* than the probiotic *S. gordonii* . Data are presented as mean ± standard deviation ( *n* = 10, ** *p* < 0.01).

The growth curves of *S. mutans* and *S. gordonii* were monitored by spectrophotometric analysis as they reached log phase at around 9 and 10 hours, respectively (data not shown). Fig. 3 shows that the viability of *S. mutan* s with the synthesized particles was lower than 60% after 8 hours of incubation at 37°C. In contrast, the viability of *S. gordonii* in the presence of the synthesized particles was higher than 80% under similar incubation condition. The statistical analysis showed that the growth inhibition of *S. mutans* by NaF@PAA@HA@polyelectrolytes@HA@PAA particles is significantly higher than that of *S. gordonii* ( *n* = 10, *p* < 0.01). This result demonstrates the selective inhibition of cariogenic pathogens versus noncariogenic probiotics by the NaF@PAA@HA@polyelectrolytes@HA@PAA particles.

### Adhesion of NaF@PAA@HA@Polyelectrolytes@HA@PAA Particles onto Tooth Enamel

To evaluate the adhesion of NaF@PAA@HA@polyelectrolytes@HA@PAA particles onto normal tooth enamels or induced white spot lesions, the enamel slides were treated with the synthesized particles for 1 day before the analysis. The concentration and atomic ratio of fluoride on enamels were analyzed by fluoride ion electrode and EDS, respectively. Table 1 shows that the fluoride concentration on the tooth enamels with artificially induced white spot lesions (72,789 ± 25,395 ppm) was significantly higher ( *n* = 5, *p* < 0.05, twofold) than the normal ones (39,763 ± 25,309 ppm). The atomic ratio of fluoride also exhibited similar trend on both tooth enamel slides (27.07 ± 10.49 vs 18.02 ± 10.81, *n* = 5, *p* = 0.08). The results revealed that NaF@PAA@HA@polyelcetrolytes@HA@PAA particles had better adhesion onto tooth enamel slides with induced white spot lesions than the normal ones.

**Table 1:** The fluoride concentration and atomic ratio of NaF@PAA@HA@polyelectrolytes@HA particles on normal tooth enamels and artificially induced white spot lesions were analyzed by fluoride ion electrode and SEM and EDS, respectively, after 1-day treatment

	Normal	White spot lesions
Fluoride concentration (ppm)	39,763 ± 25,309	72,789 ± 25,395*
Fluoride atomic (%)	18.02 ± 10.81	27.07 ± 10.49

Note: The fluoride absorbed by tooth enamel with artificially induced white spot lesions was higher that absorbed by normal tooth enamels in both the experiments. Data are presented as mean ± standard deviation. ( *n* = 5, * *p* < 0.05).

### The pH-Dependent Fluoride Release of the Prototype Toothpaste

**Fig. 4:**
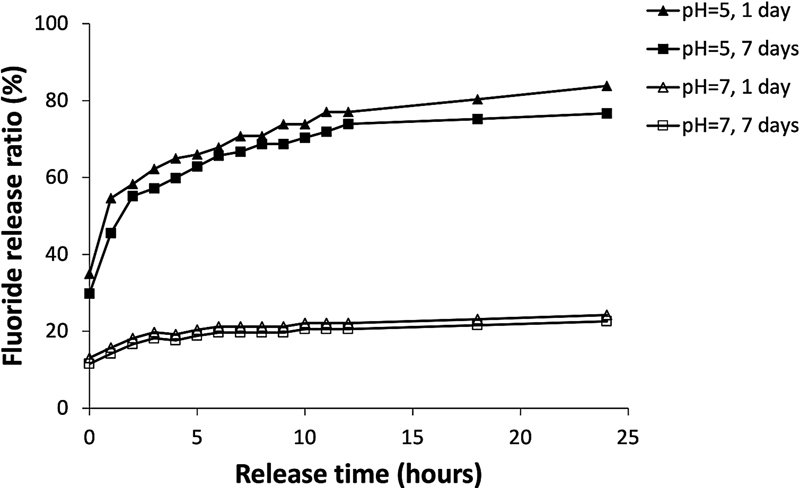
The fluoride release kinetics of the prototype toothpaste containing NaF@PAA@HA@polyelectrolytes@HA@PAA particles. After treating the enamel slide surfaces for 1 or 7 days, the fluoride concentration in the sample solutions was found to be higher in the acidic (pH = 5) than in neutral (pH = 7) environment.

The fluoride release from the prototype toothpaste containing NaF@PAA@HA@polyelectrolytes@HA@PAA particles (1%) was analyzed by fluoride ion electrode, and the results were expressed as fluoride release ratios over time, as shown in Fig. 4 . The fluoride release kinetics of the toothpaste was significantly faster and with higher fluoride release ratio in acidic environment (pH = 5) than in neutral (pH = 7). The sustained release mode was observed at both pH value. The fluoride release from the prototype toothpaste–treated enamel slide surfaces were found to be constant on day 1 and day 7 in both acidic and neutral environment. These findings indicate that the NaF@PAA@HA@polyelectrolytes@HA@PAA particles in the toothpaste have sustained environment-sensing fluoride release for long term.

### Absorption of Fluoride from the Prototype Toothpaste onto Tooth Enamel

**Fig. 5:**
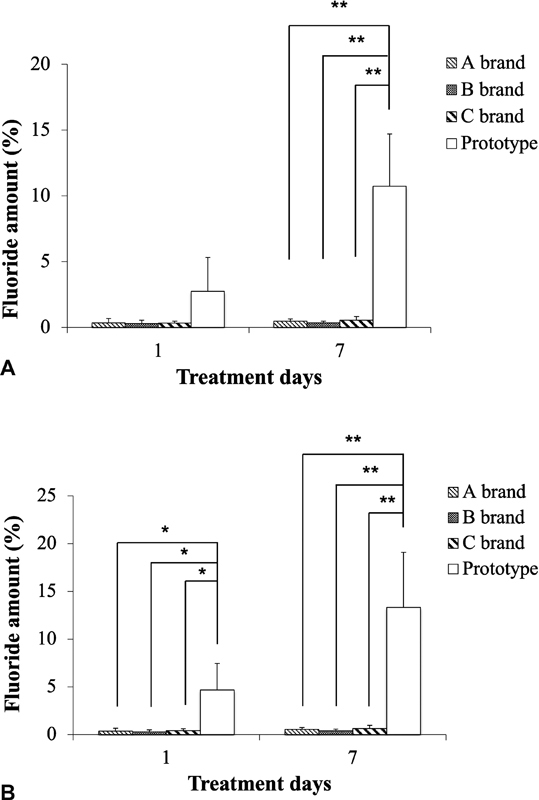
The fluoride absorption of ( **A** ) normal tooth enamel and ( **B** ) artificially induced white spot lesions after 1 or 7 days of treatments with the toothpastes. Both the normal enamel slide and the induced white spot lesion slides absorbed higher concentration of fluoride in the prototype toothpaste group than brand A, B, and C toothpastes. Data are presented as mean ± standard deviation ( *n* = 5, * *p* < 0.05, ** *p* < 0.01).

To evaluate the effect of NaF@PAA@HA@polyelectrolytes@HA@PAA particles on the enhancement of fluoride adsorption onto tooth enamel surface, as compared with three commercial toothpastes, the enamel slides were treated by the toothpaste for 1 or 7 days before analyzing by SEM and EDS. The findings of fluoride absorption on enamels analyzed by EDS are illustrated in Fig. 5A,B . Fig. 5A shows that the fluoride absorption on enamel was higher ( *n* = 5, *p* = 0.05, eight- to ninefold) for the experimental toothpaste than for all the commercial products after 1-day treatment. After 7 days of exposure, the findings were significant ( *n* = 5, *p* < 0.05, 20- to 31-fold). Fig. 5B shows that the fluoride absorption on artificially induced white spot lesions was also significantly higher ( *n* = 5, *p* < 0.05, 11- to 16-fold) for the prototype toothpaste than for all the commercial products after 1-day treatment and more significant accumulated difference ( *n* = 5, *p* < 0.01, 21- to 35-fold increase for the experimental toothpaste) was observed after 7 days of treatment. The findings indicated that NaF@PAA@HA@polyelcetrolytes@HA@PAA particles in the prototype toothpaste significantly augment the fluoride ions adsorption on tooth enamel than the commercial toothpastes.

### Anticariogenic Activities of the Prototype Toothpaste on Tooth Enamels

**Fig. 6:**
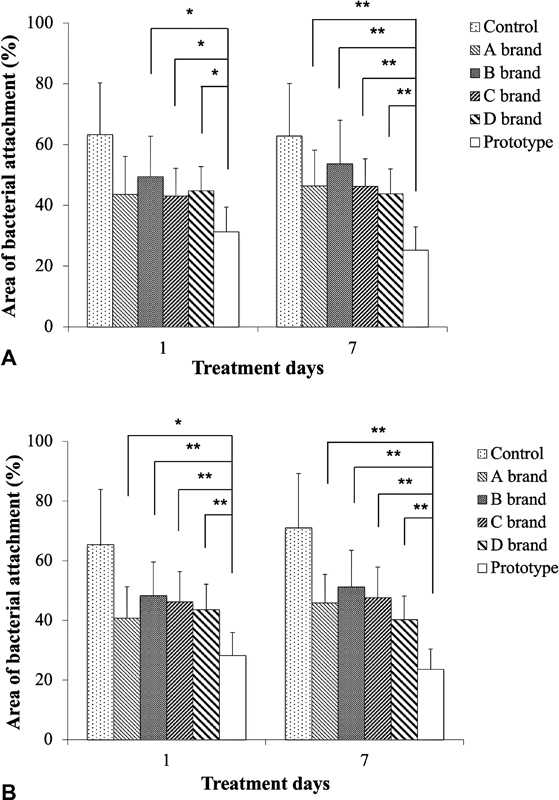
The area of *S. mutans* attached onto ( **A** ) normal tooth enamel and ( **B** ) artificially induced white spot lesions after treatment by the prototype or commercial toothpastes for 1 or 7 days. The areas on normal enamel slides and artificially induced white spot lesion slides covered by *S. mutans* after prototype toothpaste treatment were significantly less than those of the commercial toothpaste. Data are presented as mean ± standard deviation ( *n* = 5, * *p* < 0.05, ** *p* < 0.01).

The antibacterial activity of the experimental toothpastes against cariogenic bacteria *S. mutans* (OD _600_ = 0.01) was analyzed using tooth enamel slides by SEM and quantified by ImageJ software after 1- or 7-day exposure. The prototype toothpaste was compared with four selected commercial toothpastes. Fig. 6A shows that the total bacteria coverage area on tooth enamel slides treated by the prototype toothpaste for 1 day was significantly smaller (63–73%) than B, C, and D brand products ( *n* = 5, *p* < 0.05). After 7-day exposure, the results were even more significant than 1-day treatment (47–58%, *n* = 5, *p* < 0.01). Fig. 6B shows the area of bacteria growth on induced white spot lesions after being treated by different toothpastes for 1 day. The prototype toothpaste also presented significantly smaller area (58–69%) than all the commercial products ( *n* = 5, *p* < 0.05 in A brand, and *p* < 0.01 in the other three brands). In the 7-day exposure, the results were again more evident (46–59%, *p* < 0.01). These findings indicated that the prototype toothpaste is more efficient in the inhibition of cariogenic microorganisms than the commercial toothpastes.

## Discussion

Dental caries is one of the most common chronic diseases worldwide and compromises the quality of lives in all age groups. 20 Caries development is attributable to four major factors: pathogenic bacteria, teeth, sugar, and time. 21 Both pathogenic and probiotics bacteria coexist in oral cavity to maintain a balanced flora. For the initiation of caries progression, pathogen needs to access and attach to the tooth surface to form stable dental plaque where bacteria can convert carbohydrate, especially sucrose, to adhesive polymers that stabilize dental plaque and create an acidic microenvironment, which eventually dissolve the mineral components over time. 22 To break such pathognomonic cycle, the first antibacterial toothpaste containing fluoride as the major active ingredient was introduced in 1955, 7,000 years after the first tooth cleaning material documented in Egypt. 23

Fluoride treatment has been the mainstream therapy correlated with reducing the prevalence and severity of dental caries. Possible mechanisms by which fluoride prevents caries involve at least four major routes: (1) inhibition of tooth mineral dissolution by cariogenic bacteria, (2) enhancement of remineralization in caries lesions, (3) inhibition of the growth and carbohydrate metabolism of acidogenic oral bacteria, and (4) prevention of pathogens from attaching to the tooth surface. 22 24 25 26 For example, fluoride can act as an inhibitor of GTFs resulting in reduction of sugar uptake by *S. mutans* . Several studies have reported the numbers of *S. mutans* in dental plaque reduced significantly by consecutive application of fluoride at 250 to 12,300 ppm. 27 28 29 30 Such activity significantly compromises the production of adhesive polymers for the initial colonization and inhibits the bacteria growth. Fluoride also decreases cariogenic activity by reducing acid production of pathogenic bacteria and increasing acid tolerance of the tooth mineral tissues. 31

Despite the prominent antimicrobial effect of fluoride, biofilms of cariogenic bacteria significantly compromise the efficacy of fluoride, which has a limited dose range within 0.2% to 0.3% in formulation. 8 Several chemical agents were developed to supersede fluoride in toothpastes. Triclosan, for example, became the major antibacterial agent in many oral hygiene products. Triclosan inhibits bacterial growth with much lower concentration than fluoride via perturbation of their metabolic pathways, thereby preventing biofilm formation and reducing caries risk. 32 Unfortunately, most of these materials have no selectivity between pathogenic and probiotics strains, and expose the teeth to cariogenic risks upon discontinuation, as a result of imbalanced oral flora caused by collaterally damaging the probiotics. Moreover, the long-term impact of such nonselective antibacterial strategy and its well-known proneness to genotoxicity, cytotoxicity, and eco-toxicity remain unsolved. 33

According to recent clinical studies, preferential growth of probiotics in the oral microenvironment can significantly reduce the incidence of caries. 34 Probiotics can hamper the growth of pathogenic strains by adjusting the local pH, producing antimicrobial compounds, and competing space for biofilm formation. 35 36 37 Thus, it is conceivable that oral health can be benefited from rebalance flora toward probiotics. Rebalancing oral flora toward probiotics could deliver a long-term oral health to prevent a wide spectrum of pathogenic conditions. Therefore, an intelligent material that can distinguish pathogens and probiotics is the urgent clinical need in oral hygiene.

In this study, we have developed biocompatible microparticles, NaF@PAA@HA@polyelectrolytes@HA@PAA, with selectivity toward oral microorganisms. The microparticles was composed of functional core with high fluoride concentration and active attaching filaments to form a self-assembly layer on tooth surface. They also had prominent antibacterial selectivity differentially toward eradication of pathogenic *S. mutans* while sparing the probiotics such as *S. gordonii* . This selectivity was achieved by sensing the local microenvironmental pH to adjust the nanoscale pores of the polymer layer, which subsequently controls the kinetics of fluoride releasing. 38 Thus, upon triggering by the acidic microenvironment, a localized burst release of fluoride would effectively eradicate the pathogenic bacteria in situ without affecting distal bacteria due to rapid dilution by salivary fluids. In a probiotic colonized area, the relatively higher pH microenvironment would shift the microparticles to a sustained slow-release mode that would not release hazardous concentration of fluoride to damage the probiotic while maintaining a dose level capable of strengthening tooth mineral tissue against acid challenge.

With these proof-of-principle discoveries, a prototype toothpaste containing NaF@PAA@HA@polyelectrolytes@HA@PAA was made to further study the inhibition of cariogenic bacteria ex vivo. Compared with tested commercial toothpastes, the prototype toothpaste showed significantly better tooth surface fluoride absorption and more efficient inhibition of the cariogenic microorganism. Furthermore, distinct from the rest, it is the only toothpaste that promote a rebalance of oral flora toward probiotics. Despite its remarkable properties, the prototype toothpaste is recommended for adolescents and adults only. Since our research material, i.e., tested teeth enamel, was collected from adults, additional studies are needed to confirm the effects of the toothpaste on children's teeth. In addition, the fluoride burst occurs in microenvironment. It is not clear whether the microscopic high concentration of fluoride can cause any effect on children's teeth in development.

## Conclusion

In summary, we have successfully developed a smart delivery system of biointeractive microparticles that triggers differential responses between cariogenic and probiotic bacteria. Selectively eradicating pathogenic strains, the microparticles achieve a new balance of oral flora toward probiotics. Such novel delivery concept could inspire the future development of more effective products for oral preventive care.
